# Psychological Sequelae of the Station Nightclub Fire: Comparing Survivors with and without Physical Injuries Using a Mixed-Methods Analysis

**DOI:** 10.1371/journal.pone.0115013

**Published:** 2014-12-23

**Authors:** Nhi-Ha T. Trinh, Deborah L. Nadler, Vivian Shie, Felipe Fregni, Stephen E. Gilman, Colleen M. Ryan, Jeffrey C. Schneider

**Affiliations:** 1 Massachusetts General Hospital Depression Clinical and Research Program, Boston, Massachusetts, United States of America; 2 Department of Physical Medicine and Rehabilitation, Spaulding Rehabilitation Hospital, Harvard Medical School, Boston, Massachusetts, United States of America; 3 Spaulding Center of Neuromodulation, Department of Physical Medicine and Rehabilitation, Harvard Medical School, Boston, Massachusetts, United States of America; 4 Department of Social and Behavioral Sciences, and Department of Epidemiology, Harvard School of Public Health, Boston, Massachusetts, United States of America, and Department of Psychiatry, Massachusetts General Hospital, Boston, Massachusetts, United States of America; 5 Sumner Redstone Burn Center, Surgical Services Massachusetts General Hospital, Harvard Medical School, Boston, Massachusetts, United States of America, and Shriners Hospitals for Children-Boston, Boston, Massachusetts, United States of America; Univ of Toledo, United States of America

## Abstract

**Background:**

Surveying survivors from a large fire provides an opportunity to explore the impact of emotional trauma on psychological outcomes.

**Methods:**

This is a cross-sectional survey of survivors of The Station Fire. Primary outcomes were post-traumatic stress (Impact of Event Scale – Revised) and depressive (Beck Depression Inventory) symptoms. Linear regression was used to examine differences in symptom profiles between those with and without physical injuries. The free-response section of the survey was analyzed qualitatively to compare psychological sequelae of survivors with and without physical injuries.

**Results:**

104 participants completed the study survey; 47% experienced a burn injury. There was a 42% to 72% response rate range. The mean age of respondents was 32 years, 62% were male, and 47% experienced a physical injury. No significant relationships were found between physical injury and depressive or post-traumatic stress symptom profiles. In the qualitative analysis, the emotional trauma that survivors experienced was a major, common theme regardless of physical injury. Survivors without physical injuries were more likely to experience survivor guilt, helplessness, self-blame, and bitterness. Despite the post-fire challenges described, most survivors wrote about themes of recovery and renewal.

**Conclusions:**

All survivors of this large fire experienced significant psychological sequelae. These findings reinforce the importance of mental health care for all survivors and suggest a need to understand factors influencing positive outcomes.

## Introduction

One of the deadliest fires in American history, The Station nightclub fire erupted on February 20, 2003 in West Warwick, Rhode Island. Pyrotechnic sparks ignited flammable sound insulation around the stage, creating a flash fire that engulfed the club in five minutes. Of the estimated 462 attendees, over 200 were injured and 100 died [1]. Video footage of the fire depicts stampeding patrons blocking the front entrance and the ensuing pandemonium as people tried to escape the burning building. The Station Fire was an emotionally traumatic event to all survivors. In addition, a significant proportion of survivors also experienced a physical burn injury.

According to the Fifth edition of the Diagnostic and Statistical Manual, trauma involves exposure to death, threatened death, actual or threatened serious injury; or actual or threatened sexual violence by direct exposure, witnessing in person, or indirectly or through a close relative, friend, or through professional duties [2]. Psychological trauma may accompany physical trauma or exist independently of it. A life-threatening event, such as a large scale fire, can result in both physical injuries and psychological trauma, resulting in psychological sequelae (eg., post-traumatic stress disorder, major depression, anxiety disorders) in addition to impairments in occupational, functional and quality of life outcomes [3] [4] [5] [6] [7].

The Station Fire cohort all experienced the same life-threatening event, a large-scale fire, with approximately half of the sample with physical injuries and half without physical injuries, providing an opportunity to explore the effects of emotional and physical trauma on psychological outcomes. In 2013, contrary to their initial hypothesis, Schneider et al demonstrated no significant differences between survivors with and without physical injuries in the following outcomes of depressive symptoms, PTSS, or quality of life (as measured by the Beck Depression Inventory (BDI), Impact of Event Scale-Revised (IES-R), and Burn Specific Health Scale-Brief (BSHS-B), respectively) [8]. This is in contrast to prior literature on PTSS and depressive symptoms in burn survivors, thus underscoring the impact of non-physical trauma on psychological outcomes [9] [10] [11] [12].

Given this unexpected finding, we were interested in understanding in more detail the psychological sequelae experienced by the survivors. Prior evidence suggests that the presence of physical injuries after traumatic events may alter the experience of depressive or post-traumatic stress symptoms. Depressed patients with significant medical co-morbidities may experience more somatic symptoms in the context of their depressive illness [13]. Similarly, survivors with burn injuries are likely to undergo multiple medical procedures (eg., intravenous access procedures, intubation and possible tracheostomy, skin grafting surgeries with donor sites and dressing changes to achieve wound closure) [14]; these invasive medical procedures put patients at risk for high levels of PTSS [15] [16], including intrusive symptoms [17].

Finally, we used the free-response section of the survey to further understand potential differences between the two groups’ psychological experiences. Although the survey was based primarily on multiple-choice, quantitatively-based questionnaires, at the end there was an optional free-response section where survivors could comment on their personal experience in their own words. Although this was an optional section not initially intended for research purposes, our synthesis of the data from this section complements the quantitative analyses.

The purpose of this study is to further explore differences in psychological sequelae of survivors with and without physical injuries using (1) quantitative data from depressive and post-traumatic stress symptom scales, and (2) qualitative data from the free-response section of the survey. The authors hypothesize that, in contrast to survivors without physical injuries, survivors with physical injuries will report higher levels of somatic symptoms on the Somatic-Affective subscale of the BDI and higher levels of intrusive symptoms on the IES-R scale. Also, the authors hypothesize that the descriptive analysis of the free-response data will highlight the lasting physical consequences of the fire for those survivors with physical injuries as compared to those without physical injuries.

## Methods

The present study represents a more in-depth analysis of psychological outcomes, as well as a qualitative analysis of the free-response section of the survey. All survivors present at The Station nightclub on the evening of the fire on February 20, 2003 were eligible for inclusion. There were no explicit exclusion criteria.

### Recruitment

The following recruitment strategy has been detailed previously [8]. Subjects were recruited from June 2005 to October 2007 by (1) a letter from their treating rehabilitation physician, (2) survivor support group email listserve, (3) newspaper and radio advertisement, and (4) direct mailing. In the first wave of recruitment, the first three methods were utilized, as these were considered the least intrusive means of recruitment. At the time of the study, a local newspaper identified 330 likely survivors by name and hometown; the sources of information for these survivors were varied and included: survivors interviewed by the newspaper, survivors identified by other survivors, survivors identified by lawyers, survivors identified by relatives, survivors confirmed by hospitals, and survivors identified by photographers that took pictures in the nightclub [18].

In the second wave of recruitment, a search agency was used to establish definitive mailing addresses for remaining likely survivors from the initial newspaper listing. The search agency encountered numerous difficulties identifying survivors that included: incomplete versions of survivors’ names (e.g., ‘‘J. Smith’’ could have been John Smith or Jay Smith), multiple contact addresses for one potential survivor (e.g., ‘‘John Smith from Main Street’’ versus ‘‘John Smith from Center Street’’), and addresses with hometowns that differed from the newspaper listing (e.g. newspaper listed John Smith from Providence; search agency found John Smith from Portsmouth). In cases that did not have one definitive mailing address, such as the above scenarios, mailings were sent to each potential contact to attempt to reach as many survivors as possible. The mailing to these remaining likely survivors included a brief explanation of the study and study staff contact information. Survivors were invited to notify study staff if they were or were not interested in the study. Interested survivors were provided the questionnaire, which was made available by email, password-protected website, or mailed hard copy. If a completed survey was not received by two weeks, subjects received follow-up by email, phone, or mail in an effort to increase response rate. Subjects received monetary compensation for completing the survey ($25).

Written informed consent was obtained for the subjects that were recruited by letter from their treating physician. For the remainder of the study subjects, a waiver of consent was obtained from the Partners Human Research Committee. The Common Rule [19] and HIPAA Privacy Rule [20] allow an Institutional Review Board to approve a waiver of informed consent for research when specific criteria are met. Identifying data was kept separate from the rest of the data and was not used in data analysis or reporting. All study procedures were approved by the Partners Human Research Committee.

### Survey

Participants completed a 130-question multi-dimensional survey of their demographic (gender, age, race, marital status, number of children, pre-fire employment status), medical (presence and percentage of burn injury), occupational outcomes (time off work, post-fire employment status), and quality of life, as measure by the Burn Specific Health Scale-Brief (BSHS-B) [21]. Complete data on all survey variables have been previously detailed [8].

The current study focuses on the following outcomes: (1) post-traumatic stress symptoms, measured by the Impact of Event Scale – Revised (IES-R), (2) depressive symptoms, measured by the Beck Depression Inventory (BDI). The IES-R is a 22-item self-report measure that assesses subjective distress caused by traumatic events; higher scores correspond with a higher degree of PTSS. The IES-R is divided into three subscales, or domains: Intrusion, Hyperarousal and Avoidance. This assessment and its subscales have established validity and reliability [22] [23]. The BDI is a 21-question self-report inventory that assesses the existence and severity of depressive symptoms. Higher scores indicate more severe depressive symptoms. Steer et al established two domains within the BDI questionnaire: Somatic-Affective and Cognitive [11]. This questionnaire and its subscales have established validity and reliability [24] [25].

Finally, at the end of the extensive, 130-question survey, participants were given a free-response area, where they were able to put any additional comments, thoughts or observations. The free-response area followed the question: “*Is there anything else you would like to tell us that was not covered in the survey? If so, please enter the information below.”* There was no text limit for this free-response area.

### Analysis

#### Response Rates

The response rates were calculated as the ratio of the number of survivors with completed surveys to the total number of eligible survivors. Given that the newspaper listing of likely survivors was only a rough estimate of the total number of survivors and the limitations of the search agency identification process, the exact number of eligible survivors is unknown. Therefore, two response rates were calculated to provide a range [8]. For the minimum response rate calculation, eligible survivors were defined as all individuals with confirmed contact information as well as any mailings without a response; those returned because of wrong addresses were considered cases of unknown eligibility. For the maximum response rate calculation, eligible survivors were defined as only those with confirmed contact information; mailings without a response and those mailings returned because of wrong addresses were considered cases of unknown eligibility [26]. Responders that completed the survey were compared to non-responder survivors using adjusted multivariate analysis with the following variables: gender, age, and median home value by zip code. This analysis used the latter definition of eligible survivors.

#### Statistical analysis of psychological subscales

Linear regression was used to assess the relationship between physical injury and depressive and post-traumatic stress symptoms. The subscales of the Beck Depression Inventory II (cognitive and somatic-affective) and the Impact of Event Scale-Revised were examined (avoidance, hyperarousal, and intrusion). We adjusted these comparisons for the following individual characteristics: age, gender, race, marital status, number of children, pre-fire employment; and outcomes: PTSS, depressive symptoms, quality of life (BSHS-B) and employment (time off work and employment status). The following variables were dummy coded to binary variables of 0 and 1: burn injury, gender, age, race, marriage, pre-fire employment, presence of children, time off work, post-fire employment. Specifically, variables were coded as follows: burn (presence/absence), gender (male/female), race (Caucasian/not Caucasian), marriage (single, divorced, separated/married, long-term partner), pre-fire employment (full-time/part-time), presence of children (yes/no), time off work (less than six weeks/six weeks or more), and post-fire employment (full-time/part-time). The two variables age and days since fire were kept as continuous variables. The BSHS-B, BDI, and IES-R outcomes were included in the model because the outcomes are significantly correlated with each other. In the first step of modeling, univariate analyses were examined for each of the subscale outcomes with burn injury as the main independent variable. In the multivariate analysis, the simultaneous equation regression model was used for analysis. Variables considered clinically important were included regardless of their statistical significance in the univariate analysis. The above noted variables, in addition to BSHS-B, BDI, and IES-R, were then treated simultaneously and run against the continuous outcomes: cognitive and somatic depressive symptoms and hyperarousal, avoidance and intrusion PTSS. All statistical analyses were performed with STATA software, version 13 [27].

#### Qualitative Analysis

For the free-response data from the survey participants, content analysis, a systematic classification process of coding and identifying themes or patterns, was used to analyze the data. Responders were divided into two groups, those with physical injuries, and those without physical injuries. Data from each group was reviewed first separately, then together to compare themes by group. Text was separated into themes, using a Microsoft Excel database to summarize the responses from the survey recipients. Two research team members (NT and VS) independently reviewed the data, and then used the databases generated to examine the answers for themes and sub-themes reflecting the target population’s perspectives by injury group. The two research team members conducted this iterative process until all data were examined, and patterns emerged from the data that were meaningful and could be well-articulated. Recurrent themes within each content area were identified, and common themes were then grouped together into categories and summarized into tables. The two research team members then compared their analyses and came to an agreement regarding the recurrent and relevant themes into a subsequent final analysis. An independent reviewer (JCS) reviewed the data separately, examined the final analysis for consistency, and confirmed that the conclusions reached were substantiated by the data.

The recurrent, relevant themes inform the description of results; short quotes are used to report direct responses from survey participants. To illustrate the relative importance of the themes of this descriptive analysis, a “word cloud” was generated using the free response data that highlighted the most common themes as decided by the two coders (NT and VS) using Wordle online software [28], which generates word clouds that increase word size based on frequency. Word clouds and the Wordle software have been shown to be a reliable method to report and depict descriptive data [29]. Connecting words such as *the, but* and *and* were removed from the word cloud by the Wordle software. For the combined raters’ themes, themes that recurred at least four times were represented as a recurring theme. The size of the font was directly proportional to the frequency of the recurring theme [28].

#### Free-Text Responder Analysis

In addition, a quantitative analyses was completed to compare 1) responders to the free-response section with physical injuries with those free-text responders without physical injuries, and 2) within each survivor group (with and without physical injury), we compared responders to the free-response section to non-responders. Variables assessed included gender, age, race, having any children, relationship status, and median home price by zip code. For categorical variables of gender, relationship status, race, employment status, and the presence of children, Pearson’s chi-squared tests were performed. For age and median home price by zip code, t-tests was performed to see if there was a difference in mean age or median home price between groups.

## Results

### Responders

Of the 362 likely survivors, 104 survivors completed the study survey. The average number of days between the event of the fire and completion of the survey was 1280.13 days (range: 840 to 1810 days). The minimum response rate calculation included 247 eligible survivors and the maximum response rate calculation included 144 eligible survivors resulting in 42% and 72% response rates, respectively. The first wave of recruitment resulted in the identification of 120 survivors. Of these, 90 survivors completed the survey. In the second wave, a search agency identified contact information for 152 potential subjects of the remaining 210 probable survivors. Of the 24 survivors that responded to the mailing, 14 completed the survey, six responded with interest in the study but did not complete the survey and four responded that they were not interested. There were 25 returned mailings because of wrong addresses and 103 mailings without a response. Of the 104 completed surveys, 74 were by password-protected website, 30 were by mailed hard copy and none were be email. Responders and non-responders were assessed for differences in sociodemographic characteristics. Multivariate analysis showed that gender and median home price by zip code were not significantly different between groups (age, p = 0.73; home price, p = 0.22). However, gender exhibited statistically significant differences between groups, with more males in the non-responder group (p = 0.05). We therefore adjusted our results to gender as to avoid a potential effect of non-responders in our results.

### Characteristics of study population

Detailed information regarding subject recruitment has been described previously [8]. About one half of the responders sustained a burn injury as a result of the fire (47%). Characteristics between survivors with and without burn injuries were similar, except for those survivors with a burn injury were less frequently married and employed (p<0.05) (Table 1).

**Table 1:** Survey Participant Characteristics.

Category		Survivors withPhysical Injuries	Survivors withoutPhysical Injuries
**Number of subjects**		49	55
**Male**, n (%)		28 (57)	36 (65)
**Age at injury**, mean years (SD)		32.1 (6.8)	32.6 (7.5)
**Race**, n (%)			
	Caucasian	49 (98)	53 (96)
	African American	0 (0)	0 (0)
	Hispanic	1 (2)	1 (2)
	Other	0 (2)	1 (2)
**Married or long-term partner**, n (%)*		15 (31)	35 (63)
**Employment Status**, n (%)*			
	Full-time	33 (72)	50 (92)
	Part-time	7 (15)	2 (4)
	Student	4 (9)	1 (2)
	Unemployed	2 (4)	1 (2)
**Children**, n (%)		26 (53)	23 (42)
**Total body surface area burned**, n (%)			
	0–20%	29 (59)	
	21–40%	13 (27)	
	>40%	7 (14)	

### Quantitative Analysis

The primary analysis found that the survivors who sustained burn injuries from the fire had no more likelihood of experiencing Post Traumatic Stress Symptoms (PTSS) or from depressive symptoms than those without burn injuries [8]. We assessed if survivors with or without burn injuries had differences in profiles of post-traumatic stress and depressive subscale symptoms and found no significant relationships between the depressive or post-traumatic stress symptom subscales and burn injury in the multivariate analysis.

The results of the multivariate analyses are presented for the cognitive and somatic-affective subscales of the BDI in Table 2 and the intrusion, hyperarousal, and avoidance subscales of the IES-R in Table 3. For depressive symptoms (BDI), there was a statistically significant relationship with burn injury and the cognitive and somatic-affective subscales in the univariate analysis (p = 0.015 and 0.043 respectively), but not in the multivariate analysis (p = 0.68 and 0.83 respectively). For PTSS (IES-R), there was no significant relationship with burn injury and the intrusion, hyperarousal, and avoidance subscales in either the univariate or multivariate analyses (for the univariate analyses, p = 0.23, p = 0.27, and p = 0.29 respectively; for the multivariate analyses, p = 0.72, p = 0.68, and p = 0.92 respectively). In further analyses, in which outcomes were removed as covariates from the model, there continued to be no significant relationship between physical injury and either the BDI and IES-R subscales.

**Table 2:** Multivariate analyses of Beck Depression Inventory (BDI): Cognitive and Somatic-Affective Subscales.

Variable	Cognitive Subscale		Somatic –Affective Subscale		
	*β* Coefficient (95% CI)	p-value	*β* Coefficient (95% CI)	p-value	
**Burn Injury**	−0.40 (−2.36, 1.56)	0.68	−0.34 (−3.47, 2.79)	0.83	
**Gender**	−0.15 (−1.54, 1.23)	0.83	0.53 (−1.69, 2.74)	0.64	
**Age**	−0.04 (−0.15, 0.07)	0.46	0.01 (−0.19, 0.17)	0.93	
**Race**	1.49 (−2.73, 5.72)	0.48	0.86 (−5.88, 7.60)	0.80	
**Marriage**	−0.71 (−2.14, 0.72)	0.32	0.55 (−1.73, 2.83)	0.63	
**Pre-fire employment**	0.12 (−1.87, 2.12)	0.91	−2.22 (−5.41, 0.96)	0.17	
**Presence of children**	−0.25 (−1.36, 1.87)	0.76	1.72 (−0.86, 4.30)	0.19	
**Time off work**	0.95 (−1.00, 2.91)	0.33	1.89 (−1.23, 5.00)	0.23	
**Post-fire employment**	−0.53 (−2.18, 1.11)	0.52	−0.25 (−2.88, 2.38)	0.85	
**Days since fire**	−0.001 (−0.003, 0.001)	0.20	−0.001 (−0.003, 0.004)	0.72	
**IES-R**	0.08 (0.04, 0.11)	0.000	0.19 (0.14, 0.24)	0.000	
**BSHS-B**	−0.07 (−0.10, −0.04)	0.000	−0.09 (−0.13, −0.04)	0.000	
**Constant**	13.54 (7.44, 19.63)	0.000	11.56 (1.84, 21.29)	0.021	
	Cognitive Subscale (*R^2^* = 0.62) F (12, 61) = 8.23		Somatic-Affective Subscale (*R^2^* = 0.71) F (12, 61) = 12.26		

Note: *β* coefficients are unstandardized.

**Table 3:** Multivariate analysis of Impact of Event Scale-Revised (IES-R): Intrusion, Hyperarousal, and Avoidance Subscales.

Variable	Intrusion Subscale		Hyperarousal Subscale		Avoidance Subscale	
	*β* Coefficient (95% CI)	p-value	*β* Coefficient (95% CI)	p-value	*β* Coefficient (95% CI)	p-value
**Burn Injury**	−0.88 (−5.70, 3.95)	0.72	−0.83 (−4.78, 3.12)	0.68	0.27 (−4.80, 5.34)	0.92
**Gender**	1.85 (−1.24, 4.94)	0.24	2.10 (−0.44, 4.63)	0.10	1.76 (−1.49, 5.01)	0.28
**Age**	0.10 (−0.16, 0.36)	0.44	0.01 (−0.18, 0.24)	0.79	0.15 (−0.12, 0.42)	0.27
**Race**	−4.76 (−14.28, 4.76)	0.32	−2.25 (−10.68, 4.92)	0.46	−3.98 (−13.99, 6.03)	0.43
**Marriage**	0.29 (−2.96, 3.53)	0.86	−0.66 (−2.16, 3.15)	0.71	−0.31 (−3.72, 3.01)	0.86
**Pre-fire employment**	4.20 (−0.60, 9.00)	0.09	0.52 (−3.56, 4.31)	0.85	2.41 (−2.64, 7.45)	0.34
**Presence of children**	−0.94 (−4.63, 2.76)	0.61	0.59 (−3.80, 2.25)	0.61	−2.04 (−5.92, 1.85)	0.30
**Time off work**	−1.33 (−6.15, 3.49)	0.58	0.20 (−4.10, 3.79)	0.94	−2.82 (−7.88, 2.24)	0.27
**Post-fire employment**	−2.22 (−6.16, 1.72)	0.26	0.24 (−3.34, 3.12)	0.95	−0.13 (−4.28, 4.01)	0.95
**Days since fire**	0.0001 (−0.002, 0.007)	0.96	0.002 (−0.003, 0.004)	0.71	0.003 (−0.002, 0.007)	0.26
**BDI**	0.69 (0.50, 0.88)	0.000	0.58 (0.40, 0.71)	0.000	0.40 (0.20, 0.60)	0.000
**BSHS-B**	0.02 (−0.05, 0.10)	0.55	0.02 (−0.06, 0.07)	0.86	0.01 (−0.07, 0.09)	0.80
**Constant**	−1.70 (−16.89, 13.48)	0.82	−1.13 (−13.76, 11.11)	0.83	−4.09 (−20.05, 11.87)	0.61
	Intrusion Subscale (*R^2^* = 0.63) F (12, 59) = 8.21		Hyperarousal Subscale (*R^2^* = 0.65) F (12, 59) = 8.94		Avoidance Subscale (*R^2^* = 0.37) F (12, 59) = 2.84	

Note: *β* coefficients are unstandardized..

### Qualitative Analyses

**Figure 1:**
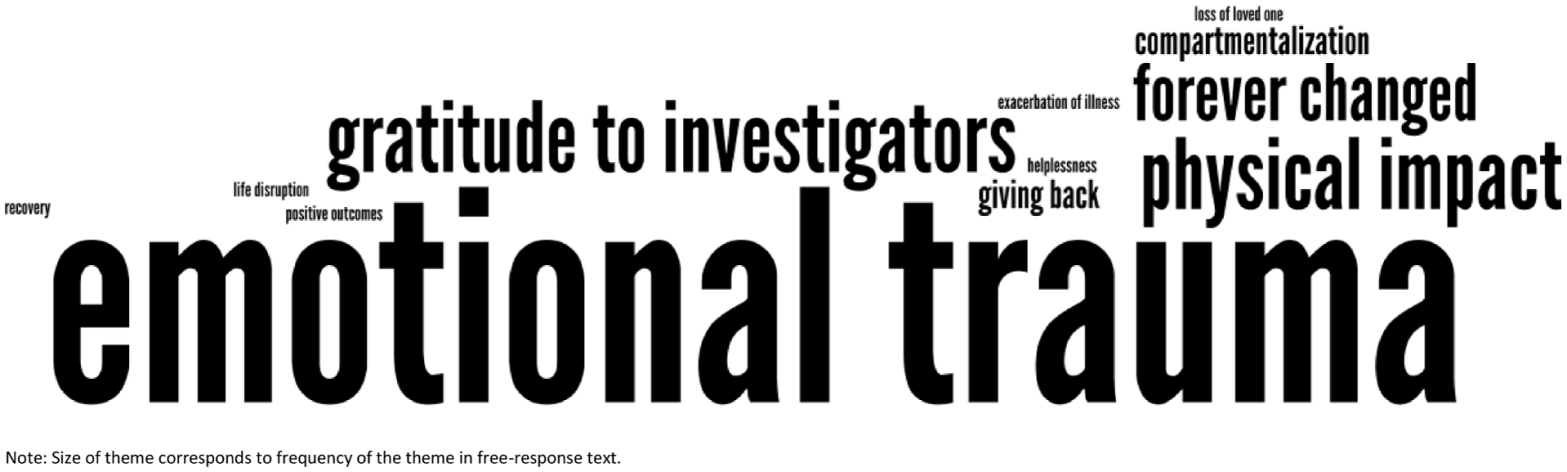
Word Cloud of Free-text Response Themes: Survivors with physical injuries. The word cloud contains themes prominent in the free-text responses of survivors with physical injuries. The size of each theme corresponds to the frequency the theme emerged in the free-responses.

**Figure 2:**
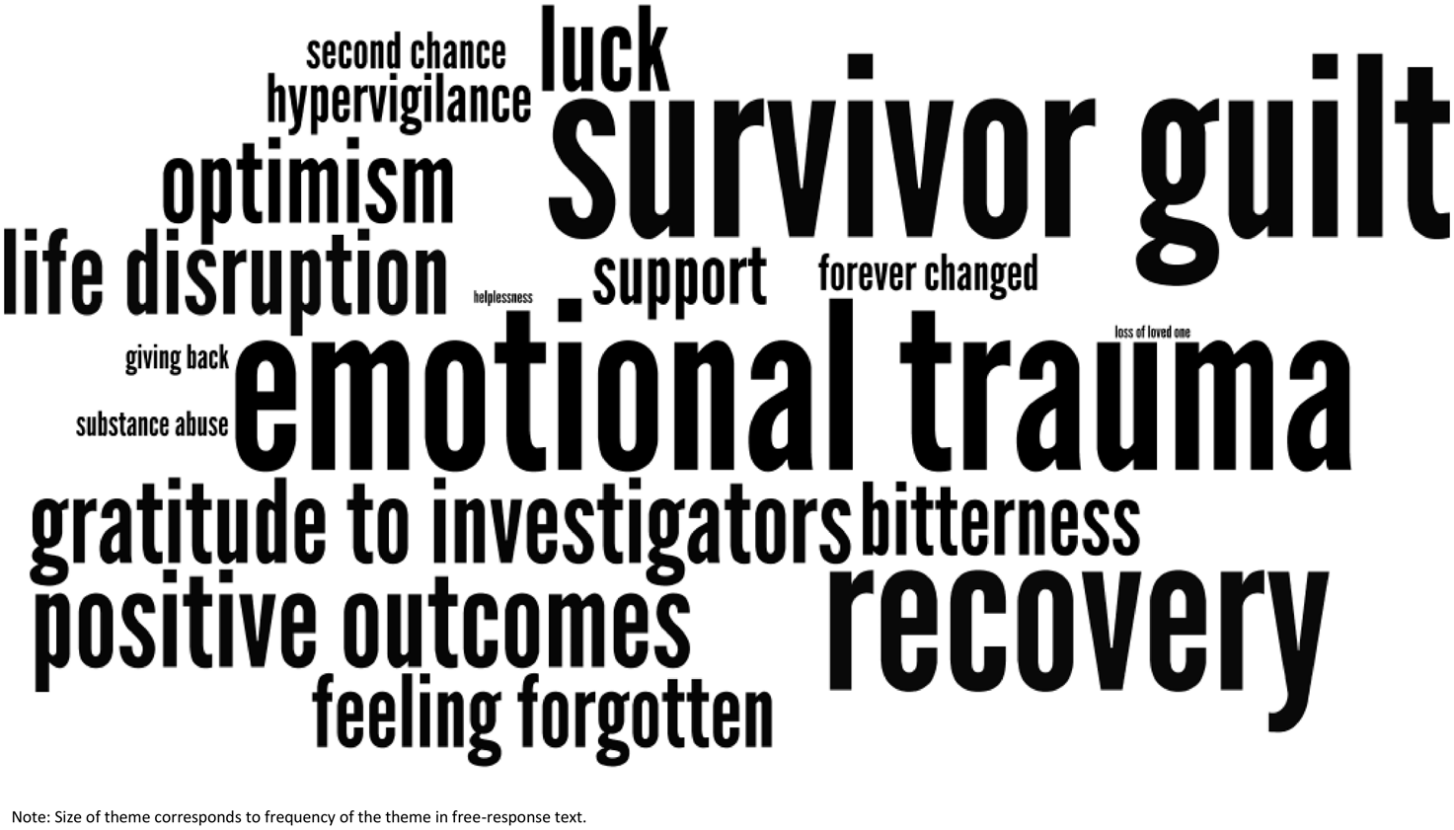
Word Cloud of Free-text Response Themes: Survivors without physical injuries. The word cloud contains themes prominent in the free-text responses of survivors without physical injuries. The size of each theme corresponds to the frequency the theme emerged in the free-responses.

Of the 104 survivors that completed the study survey, 41 chose to write responses in the free response section (39%): of those, 17 respondents experienced physical injuries, and 24 did not. The emotional trauma that survivors experienced was a major, common theme, whether or not a physical injury was involved. Table 4 lists the recurrent themes and their frequency by survivor group. Figs. 1 and 2 are Wordle word clouds generated from the current themes for survivors with and without physical injuries respectively. Below is a detailed summary of the descriptive analysis.

**Table 4:** Survivor Free-Text Response Themes by Frequency.

	Frequency of Themes	
Themes	Survivors withphysical injuries	Survivors withoutphysical injuries
Emotional trauma, Forever changed	19	17
Recovery, Positive outcomes	2	21
Survivor guilt, Bitterness	0	21
Life disruption, Physical impact, Loss of loved ones	11	8
Support, Optimism, Giving back	2	15
Feeling forgotten, Helplessness, Compartmentalization	3	7
Luck, Second chance, Hypervigilance	0	14
Gratitude to investigators	5	7
Substance abuse, Exacerbation of illness	1	2

#### Survivors with physical injuries

Of the 17 RI fire survivors with physical injuries, the emotional trauma they endured was the most common theme that emerged, either trauma directly stemming from the fire itself, or from dealing with their physical injuries. This group also noted the lasting physical impact of their injuries and the challenges of life disruption they faced as they attempted to return back into their daily routines after the event. As a direct result of their injuries, many responders commented on themes of ensuing financial stress as well as disruption to their daily lives, such as inability to work. Though this group of survivors noted how they felt they were forever changed, they also responded with positivity in their desire to give back and expressed gratitude towards investigators for conducting the survey.

#### Survivors without physical injuries

For the 24 survivors who did not experience a physical injury, themes of survivor guilt and recovery were the most frequent themes, closely followed by emotional trauma. Regarding survivor guilt, many of this group described feelings of regret about not being able to help others escape, or guilt that they were left “unscathed” as they reflected on others, including loved ones, who were physically injured or lost in the fire. Many responses reflected themes of self-blame and bitterness over the loss of others. Yet, many considered themselves fortunate, and even lucky, to have escaped the fire physically unscathed, and positive themes of awareness, hope, and perseverance also emerged as responders noted their optimism for the future and the positive changes they have made to their current lifestyle.

Many survivors without physical injuries noted the lasting traumatic impact of imprinted images from the fire, where they were “scarred” emotionally by the event even though they were not physically harmed. As a result, survivors were forever changed, emotionally, physically, occupationally, personally: “I am not the same person I was four years ago.” Perhaps as a result, a few responders alluded to using alcohol, smoking, or other “self-destructive” behaviors to forget about what had happened to them. Further, after the event, survivors without physical injuries developed hypervigilance to their surroundings, fear of crowds, and a newfound awareness in fire prevention safety. Finally, many survivors without physical injuries felt no justice was served, or individually they were not given enough resources or support along the way. Some were angry that the survivors have been “forgotten” or “made victims” from the lawsuits that followed. Although some felt they faced significant obstacles before the fire, they acknowledged that the trauma’s impact magnified their challenges.

#### Comparison of survivor groups

More study participants who did not sustain physical injuries opted to comment in the free-response section than those who sustained physical injuries. What is interesting is that both groups endured significant emotional trauma that they describe as scarring. Furthermore, those with physical injuries mentioned the lasting impact of emotional trauma more often than the physical trauma of the event. Survivors gave graphic descriptions of the emotional trauma and psychological sequelae of what they witnessed, including the unforgettable nature of this trauma, in which many felt helpless to intervene. Many survivors spoke of how these images have continued to haunt them. The trauma of experiencing and re-experiencing the event affected the survivors significantly, which they voice as a lasting impact of the fire.

In addition, while the responses from survivors with physical injuries depicted themes of physical impact, disruption, and changes of their daily lives as a result from their injuries, responses from survivors without injuries depicted more predominantly, themes of helplessness, survivor guilt, self-blame, and bitterness. In particular, survivors without physical injuries commented on their frustration with the lack of resources for their recovery from the traumatic event, as well as with the emotional pain knowing that they had survived while others had not. One responder, in particular, noted feeling that they were not considered “a true survivor” because they “don’t have the physical scars.” Certain survivors without physical injuries used alcohol or other substances as coping mechanisms after the event. A few survivors without physical injuries used support from their friends, family, and work or even moved away from to cope with the emotional trauma.

However, in spite of the post-fire challenges the survivors described, many survivors in both groups wrote about themes of recovery and renewal, including gratitude for the support they received from their families and friends, a desire to give back, and feelings of perseverance towards the future. Themes of resilience and recovery emerged despite significant obstacles of physical and emotional trauma. One survivor talked about confronting their guilt head on; another was surprised that the survey did not ask if anything positive came out of the experience, and yet another remarked that their awareness of safety was heightened from the event. Though there was trepidation at how the survey would be used, many respondents were also grateful to the investigators for paying attention to this issue, and some were curious about what the results of the survey would be. Survivors also voiced how lucky and how grateful they felt to have a “very rare second chance in life.” Many responders wrote that the fire had given them a new perspective on life, and allowed them to achieve sobriety or some type of spiritual awakening, “*To really enjoy life and embrace every opportunity to positively change and grow in life.”* A number of people wrote about the steps they were taking or had taken to recover and/or renew themselves after the fire, and acknowledged the good fortune of being able to lean on the “*wonderful support group”* of others.

#### Free-Text Responder Analysis

We performed a responder analysis, comparing responders to the free-response section with physical injuries with those free-text responders without physical injuries. Also, we compared responders to the free-response section to non-responders within each survivor group (with physical injury and without physical injury). Between the groups of survivors with and without physical injuries who responded to the free-response section, there were no significant differences age, race, employment status, gender, relationship status, presence of children, or median home price by zip code. Within each group of survivors (with physical injuries as and without physical injuries), there were no significant differences age, race, employment status, gender, in relationship status, or median home price by income between responders and non-responders. However those survey participants with both physical injuries and children were less likely to have responded to the free-response section than those with physical injuries but without children (Pearson’s chi-squared test, p = 0.03).

## Discussion

Overall, this analysis represents a more in-depth portrait of the psychological sequelae experienced by Rhode Island nightclub fire survivors with and without physical injuries. Our two complementary analyses confirm and deepen the initial findings from Schneider et al that the fire survivors with and without physical injuries presented with similar depressive and PTSS, suggesting that non-physical trauma is the primary determinant of these outcomes [8]. Contrary to our primary hypothesis, statistical tests used to analyze subscales of the BDI and the IES-R revealed no differences between survivors with and without physical injuries. The analyses reveal that survivors with and without physical injuries do not differ significantly in the cognitive or somatic-affective dimension of depressive symptoms, or in the hyperarousal, intrusion or avoidance dimensions of post-traumatic stress symptoms. Furthermore, a descriptive analysis probing into the free-text responses of survivors yielded a compelling portrait of the depth of psychological consequences of this trauma in those with and without physical injuries. This is further evidence to suggest that the non-physical injuries resulting from the event itself were the primary root of these psychological outcomes.

The descriptive analysis of the free-response section of the survey also confirms these results. Survivors struggled with the lasting emotional impact of the fire, as well as the impact on their psychological health. Physical injuries though present, were not the primary focus of those with physical injuries; the focus was on emotional struggles, obstacles faced, support of family and friends, and on recovery. In light of these findings, there is an increased need for long-term psychosocial follow up for survivors of disasters, both with and without physical trauma.

It is especially interesting that recovery, renewal and positive outcomes emerged from the free-response section, as the survey was not designed to investigate this type of outcome. There is a growing interest in post-traumatic resilience and post-traumatic growth, which can result from devastating, catastrophic events [30]. Though there may be a tendency to look to survivors of a traumatic event as victims of a tragedy, future research into long-term outcomes should focus on factors contributing to survivor resiliency and positive outcomes in the face of tragedy.

There are a few limitations to the study. One limitation is the cross sectional design. Because subjects completed the questionnaire at different points in time, a comparison of long-term outcomes is affected by the prolonged recruitment period. Additionally data was obtained directly from participants by a self-report questionnaire, potentially introducing a reporting bias. However, this form of data collection was selected in order to include survivors not treated by the medical system and at long-term follow-up. Also, a minority of probable survivors completed the survey introducing a potential selection bias, and further, a sub-group of those respondents chose to contribute to the free-response survey. Still, responder and non-responder analysis to the survey as a whole demonstrated no significant differences in age and socioeconomic status (median home value by zip code). Responder analysis on a variety of demographic variables revealed that survey participants who contributed to the free-response section did not differ statistically significantly from those who did not. The only statistically significant difference found was that survey respondents with physical injuries and with children were less likely to have responded to the free-response section than those with physical injuries but without children, which may speak to time pressures limiting survey recipients with children. Furthermore, there were significantly more female than male responders; historically men exhibit lower survey response rates than women [31]. Finally, the analyses contained in this manuscript are secondary, retrospective in nature, and potentially less statistically rigorous: quantifying subscales of psychological questionnaires, as well as descriptively summarizing free-text responses. In addition, no information was gathered on the impact of this event on family members, who may also have experienced significant life changes as a result of this event, both social and economic. However, in spite of these potential limitations, these analyses deepen our understanding of the contribution physical injury and emotional trauma have on the psychological health of these survivors.

Survivors of The Station nightclub fire experienced significant psychological sequelae; at the same time, they describe inspiring stories of hope and recovery. As described by many survivors, even as their physical injuries have healed, it is the internal scarring from this traumatic event that remains. This experience underscores the lasting emotional impact of the fire and at the same time, potential for healing in this community. These findings suggest a need to understand individual factors influencing positive outcomes for fire survivors. The findings also highlight the need for long-term psychological support and longitudinal follow-up for all survivors.
